# Childhood Differences in Healthcare Utilization Between Extremely Preterm Infants and the General Population

**DOI:** 10.3390/children12080979

**Published:** 2025-07-25

**Authors:** Kareena Patel, Thomas R. Wood, David Horner, Mihai Puia-Dumitrescu, Kendell German, Katie M. Strobel, Krystle Perez, Gregory C. Valentine, Janessa B. Law, Bryan Comstock, Dennis E. Mayock, Patrick J. Heagerty, Sandra E. Juul, Sarah E. Kolnik

**Affiliations:** 1 School of Medicine, University of Washington, Seattle, WA 98195, USA; 2 Department of Pediatrics, Division of Neonatology, University of Washington, Seattle Children’s Hospital, Seattle, WA 98195, USAgcvalent@uw.edu (G.C.V.); janessal@uw.edu (J.B.L.);; 3 Center on Human Development and Disability, University of Washington, Seattle, WA 98195, USA; 4 COPSAC, Copenhagen Prospective Studies on Asthma in Childhood, Herlev and Gentofte Hospital, University of Copenhagen, 2100 Copenhagen, Denmark

**Keywords:** healthcare utilization, extremely preterm, general population

## Abstract

**Background/Objective(s):** Post-discharge clinical needs of extremely preterm (EP) infants are not well defined. The aim of this study is to evaluate healthcare utilization after discharge in infants born EP and compare it to the general pediatric population. **Methods:** This study involved a post hoc analysis of infants born 24-0/7 to 27-6/7 weeks’ gestation enrolled in the Preterm Erythropoietin Neuroprotection (PENUT) Trial who had at least one follow-up survey representing their course between 24 and 60 months of age. The results were compared to the general population data from the Kids’ Inpatient Database, Nationwide Emergency Department Sample, and National Health and Nutrition Examination Survey. **Results:** Maternal, infant, and hospitalization characteristics for PENUT infants who survived to discharge (n = 828) compared to those with follow-up (n = 569) were similar except for race and maternal age. Overall, EP infants had an overall lower rate of ED visits (31% vs. 68%) but a higher rate of hospitalizations (11% vs. 3%). EP infants were less likely to go to the ED for gastrointestinal (5% vs. 12%) and dermatologic (1% vs. 6%) concerns but more likely to go to the ED for procedures (7% vs. <1%). EP infants had a higher rate of medication use (56% vs. 14%) in all categories except psychiatric medications. **Conclusions:** While EP infants had higher rates of specialty healthcare utilization relative to the general pediatric population, they were less likely to visit the ED overall, particularly for common concerns in this age range. This may reflect improved access and navigation of the healthcare system by EP caregivers.

## 1. Introduction

Advances in neonatology have improved morbidity and mortality in some of the most fragile infants. However, most of the literature concerning preterm infants is focused on in-hospital outcomes. A review by Kang et al. in 2021 emphasized the need for further research on the long-term outcomes of prematurity to inform appropriate follow-up care after neonatal intensive care unit (NICU) discharge [1].

Existing studies have revealed a discrepancy in healthcare utilization and expenditures between preterm and term infants within the first two years of life. Levin et al. showed significantly higher medication cost and use for preterm as opposed to term infants [2]. Similarly, odds of rehospitalization after NICU discharge and the rates of ED visits were higher in infants born preterm within the first two years of life [3,4]. Overall, there is an inverse relationship between healthcare utilization and gestational age (GA) within the first 2 years of life in preterm as opposed to term infants, with the highest utilization observed in infants born <29 weeks [2,3,4,5,6]. However, it is unclear whether healthcare utilization differences in term versus preterm infants persist beyond the first 2 years of life.

Higher healthcare utilization in preterm as opposed to term infants is likely driven by a variety of factors [6,7]. Neonatal comorbidities common in extremely preterm (EP) infants, such as bronchopulmonary dysplasia (BPD), necrotizing enterocolitis (NEC), and hydrocephalus, explain some but not all of the discrepancy noted to date in healthcare resource utilization in early childhood after discharge [4,6,7,8]. In a study of 892 preterm infants, ~50% with frequent clinic visits and prescriptions were described in patients who did not have NICU courses complicated by common morbidities of prematurity [6]. Other factors such as medical equipment needs [9,10] and sociodemographic characteristics including parental race, ethnicity, and insurance status [5,11] have been shown to be associated with increased readmission rates. These findings suggest that not all follow-up differences can be attributed to in-hospital comorbidities.

In this cross-sectional analysis, we will examine the healthcare utilization in EP infants enrolled in the Preterm Erythropoietin Neuroprotection (PENUT) Trial from 24 months to 60 months of age. Our objectives are to characterize hospitalizations, ED visits, and medication use in EP infants throughout early childhood years and compare these patterns to those of the general population. Our secondary aim is to determine potential drivers and associations between healthcare utilization in this preterm cohort versus the general population, using data from the Agency for Healthcare Research and Quality’s 2019 Kids’ Inpatient Database (KID), 2019 National Emergency Department Sample (NEDS), and 2019 National Health and Nutrition Examination Survey (NHANES), to elucidate gaps or needs in resource allocation, access, or education.

## 2. Methods

### 2.1. Study Population

This is a post hoc analysis of the PENUT Trial, a randomized, placebo-controlled, double-blinded, multicenter study that examined the potential effect of erythropoietin on neurodevelopmental impairment at 22–26 months’ corrected age [12]. The PENUT Trial enrolled 941 infants born 24-0/7 to 27-6/7 weeks’ gestation from 19 sites in 30 NICUs throughout the United States between December 2013 and September 2016. Infants were randomized to treatment with erythropoietin or placebo within 24 h of birth. Exclusion criteria included life-threatening anomalies, chromosomal anomalies, disseminated intravascular coagulopathy, twin-to-twin transfusion, polycythemia, hydrops fetalis, and known congenital infection. This current study included all infants who survived to discharge and had at least one follow-up between 24 and 60 months.

Parental consent was obtained for enrollment in the PENUT Trial and for use of de-identified data in future secondary and post hoc studies. The PENUT Trial was registered with the Food and Drug Administration (IND#12656) and ClinicalTrials.gov (NCT01378273) and was approved by each site’s institutional review board.

### 2.2. Data Collection

Neonatal and NICU hospitalization data were collected prospectively during the initial hospital stay. Maternal demographic information was self-reported. Neonatal comorbidities included severe BPD (requiring high-flow nasal cannula, continuous positive airway pressure, or mechanical ventilator support at 36 weeks post-menstrual age), severe IVH (grade 3 or 4), severe retinopathy of prematurity (ROP, stage 4 or 5 requiring laser surgery or bevacizumab therapy), and severe NEC (stage 2b or 3 Bell criteria). After discharge, infants underwent phone follow-up at 30, 36, 42, 48, 54, and 60 months’ chronological GA. At each follow-up phone encounter, data was collected from families regarding hospitalizations, ED and urgent care visits, and medication use over the prior six months. Therefore, our study captured healthcare utilization between 24 and 60 months’ chronological GA post-NICU discharge. Standardized parental intake forms were used by PENUT follow-up staff that included information on hospital admissions, ED visits, and medications, which were reviewed by our team and assigned a corresponding ICD-10 code, category, and/or generic medication name.

The incidence of hospitalizations for children aged 24–60 months and emergency department visits was obtained from the Centers for Disease Control Health, United States 2019 annual report and National Hospital Ambulatory Medical Care 2019 Survey, respectively. These data representing the general population were compared to the incidence of hospitalizations and emergency department visits among children in the PENUT cohort.

General population data on reasons for hospitalizations and ED visits for pediatric patients from 24–59 months were collected from the 2019 KID and 2019 NEDS databases, respectively. KID and NEDS are the largest publicly available pediatric inpatient discharge and ED databases in the United States, respectively. Data from KID includes a sample of 80% of pediatric discharges from non-rehabilitation hospitals in participating states. Data from NEDS is based on a 20% stratified cluster sample of hospital-based EDs in participating states. Hospitals are randomly sampled and stratified by geographic location, trauma center designation, urban–rural location, teaching hospital status, and hospital ownership. All ED visits in each cluster were included in this study. Hospital discharge and ED visit diagnoses and procedures from the KID and NEDS databases were coded according to the International Classification of Diseases, Tenth Revision (ICD-10).

General population data on medication use were collected from the 2019 National Health and Nutrition Examination Survey (NHANES), a nationally representative cross-sectional survey of the civilian, noninstitutionalized United States population. Data for pediatric patients from 24–59 months was extracted from the NHANES Dietary Supplements and Prescription Medication Use questionnaire. For both the PENUT cohort and the general population, vitamins, supplements, and medications used in acute settings, such as antibiotics and pain medication, were excluded.

### 2.3. Statistical Analysis

Descriptive statistics were calculated for PENUT cohort characteristics with analysis adjusted for the factors used to stratify randomization (recruitment site, single or multiple birth, and gestational age) [12]. The PENUT cohort that survived to discharge was compared to the follow-up cohort using *t*-tests and chi-squared tests for continuous and categorical variables, respectively. Significance was denoted with *p* < 0.05.

We filtered the KID and NEDS data by including children aged 24 mo to 59 mo. We then grouped the data by admission to consider each hospital admission individually. If an admission had a diagnosis code but no procedure codes, we included the diagnosis code. If procedure codes were present, we included all procedure codes. This ensured that records were not duplicated with conflicting diagnosis and procedure codes. Next, we categorized the ICD-10 codes into predefined categories, based on primary organ system affected (e.g., respiratory) and/or type of admission (e.g., procedural). We further categorized ICD-10 codes into subcategories of each organ system (e.g., reactive airway disease subcategory under the respiratory category) to aid in our analysis. We calculated the percentage of admissions that fell into each category and diagnosis by matching the ICD-10 codes from the cleaned dataset with the predefined categories. We applied the same categories and diagnoses to the caregiver-reported data from the PENUT cohort. Finally, we calculated both the total count and cumulative incidence of all admissions for each category and diagnosis by aggregating all data across all age groups, including repeat encounters. To compare the KID data to the PENUT cohort for specific categories, we performed a chi-squared test to determine if there were significant differences between the two cohorts. The *p*-values for each category were calculated using a proportion test, which helped identify categories with statistically significant differences. Medications were categorized by drug type (Supplementary Table S2). The percentage and count of medications in each category were determined for both the PENUT and NHANES cohorts. A chi-squared test was conducted to compare medication use between the two groups. All analyses were performed using R version 4.4.0 in the RStudio environment. Comorbidity burden, particularly conditions related to prematurity or neonatal complications, was assessed within the EP cohort (Supplementary Table S2). However, comparable comorbidity data are not available in NHANES 2019, preventing adjustment for these factors when comparing medication use between cohorts.

## 3. Results

### 3.1. Cohort Characteristics

Of the 941 infants enrolled in the PENUT Trial, 828 survived to discharge and 569 had at least one follow-up phone survey between 24 and 60 months. Follow-up rates varied by site between 14 and 85% of enrolled infants. Of the infants with follow-up, healthcare utilization data were available for 63 (11%) at 24 months, 138 (24%) at 36 months, 226 (38%) at 42 months, 304 (53%) at 48 months, 362 (64%) at 54 months, and 362 (64%) at 60 months. Infants in the follow-up cohort were less likely to have a mother who identified as Black compared to those who survived to discharge (*p* = 0.009). Additionally, maternal age was slightly greater by 0.5 years in the follow-up cohort compared to the cohort that survived to discharge. The numbers of infants with exposure to erythropoietin and all other maternal, neonatal, and NICU hospitalization data were statistically similar between the two groups (Table 1).

The general population sample included 166,323 hospitalizations, 1,102,680 ED visits, and 3600 patients surveyed regarding medication use. Racial and ethnic demographic data for the general population samples can be found in Supplementary Table S1. To allow appropriate comparison, PENUT data were recoded to make Hispanic a separate category to align with the other datasets.

The proportion of non-Hispanic white individuals included in the analysis across cohorts was significantly different (*p* < 0.001), with PENUT having the highest non-Hispanic white and NHANES having the lowest non-Hispanic white proportion of participants.

### 3.2. Hospitalizations and Emergency Room Visits

**Figure 1:**
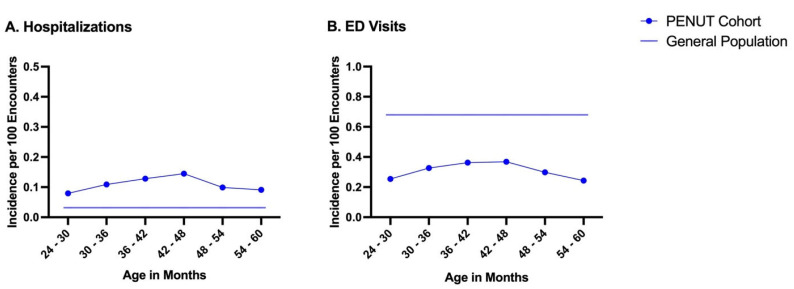
(**A**). Incidence of hospitalizations in the PENUT cohort by 6-month intervals compared to the general population. Average hospitalizations per 100 encounters: 11 compared to 3 in the general population. (**B**). Incidence of ED visits in the PENUT cohort by 6-month intervals compared to the general population. Average ED visits per 100 encounters: 31 compared to 68 in the general population. General population data were obtained from the Centers for Disease Control 2019 survey and represent aggregate hospitalization rates in children and ED visit rates in children aged 24–60 months [13,14].

EP infants had fewer overnight hospitalizations (n = 152) compared to ED visits (n = 402). The incidence of hospitalizations and ED visits in the PENUT cohort compared to the general population is illustrated in Figure 1. The average incidence of hospitalizations per 100 encounters in children 24–60 months of age was higher in the EP population compared to the general population (11% vs. 3%); however, the general population had more ED visits (31% vs. 68%).

Respiratory-related disease was the most common reason for hospitalizations and ED visits in both the PENUT cohort and the general population (Table 2 and Table 3). The most prevalent concerns in both groups were viral upper respiratory infection, bronchiolitis, and pneumonia. Among the encounters reported in the PENUT cohort, EP infants had a similar rate of respiratory hospitalizations but a higher rate of respiratory ED visits. EP infants had an almost 4-fold higher rate of ED visits for reactive airway disease compared to the general population (Table 3; 12% vs. 3% of ED visits).

Procedural encounters were more common in the PENUT cohort (hospitalizations associated with a procedure: 34% in EP infants, 12% in the general population; ED visits associated with a procedure: 7% in EP infants, <1% in the general population). In particular, EP infants were more likely to be hospitalized for tracheostomy malfunction, revision, or removal (5% vs. <1%, *p* < 0.0001), ear, nose, and throat procedures (12% vs. 3%, *p* < 0.001), gastrostomy or nasogastric tube placement, malfunction, or infection (3% vs. 1%, *p* < 0.009), and hernia repair, hip dislocation, or talipes equinovarus (4% vs. 1%, 0.009).

EP infants were also more likely to visit the ED for neurologic concerns overall (7% vs. 2%, *p* < 0.001). Specifically, EP infants had a higher proportion of ED visits (<1% vs. 0%, *p* < 0.001) and hospitalizations (1% vs. <1%, *p* < 0.001) attributed to apnea and a higher proportion of ED visits attributed to seizures or possible seizures (6% vs. 2%, *p* < 0.001).

Compared to the general population, EP infants had a lower proportion of hospitalizations for hematology/oncology concerns, hospitalizations and ED visits for dermatologic concerns, and ED visits for foreign bodies. Additionally, EP infants had a lower proportion of ED visits for infectious diseases, with a particularly lower proportion of ED visits for viral (non-respiratory) syndromes and hospitalizations for sepsis or meningitis. EP infants also had lower rates of ED visits for gastrointestinal concerns, with particularly lower rates of dehydration and gastrointestinal infections/inflammation.

### 3.3. Medication Use

Within the PENUT cohort, the highest rate of any medication use was for infants with two comorbidities. A greater number of comorbidities (3+) was not associated with a higher rate of any medication use. Of the individual comorbidities, 229/374 (61%) of infants with severe BPD, 15/25 (60%) of those with severe NEC, 40/62 (65%) of those with severe IVH, 33/50 (66%) of those with severe ROP, and 16/30 (53%) of those with hydrocephalus had at least one documented medication used.

**Figure 2:**
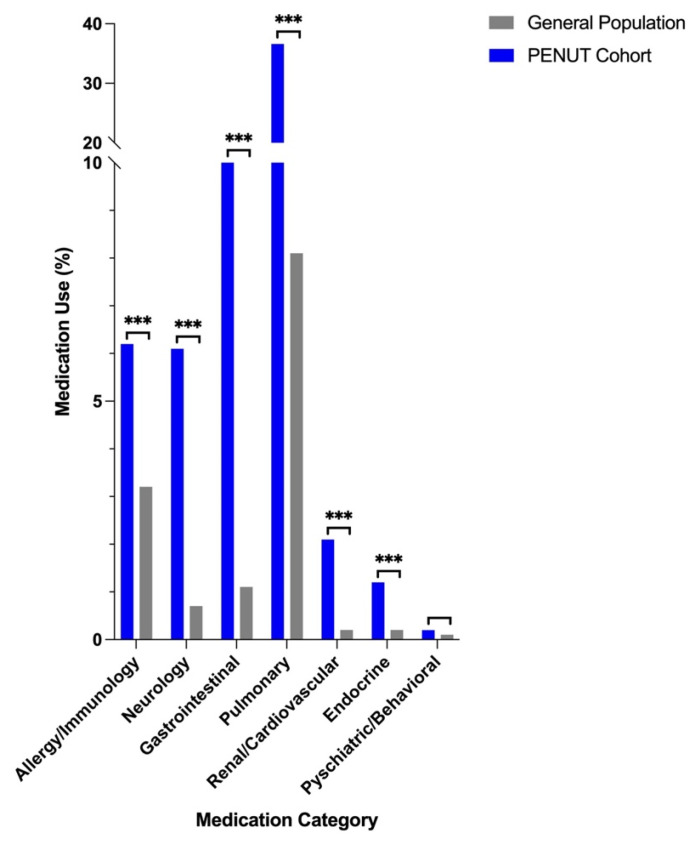
Medication use by category in PENUT participants with follow-up between 24 and 60 months and in the general pediatric population between ages 24 and 59 months: allergy/immunology (6.2% vs. 3.2%), neurology (6.1% vs. 0.7%), gastrointestinal (10.9% vs. 1.1%), pulmonary (36.6% vs. 8.1%), renal/cardiovascular (2.1% vs. 0.2%), endocrine (1.2% vs. 0.2%), psychiatric/behavioral (0.2% vs. 0.1%). General population data is from the 2019 National Health and Nutrition Examination Survey (NHANES). *** = *p* ≤ 0.001 by chi-squared test.

EP infants had a 4-fold higher likelihood of reporting any medication use compared to children in the general population (56% of PENUT subjects vs. 14% of general population subjects, *p* < 0.001). Additionally, EP infants had a higher likelihood of reporting medication use in every medication category except psychiatric and behavioral (Figure 2, Supplementary Table S2).

The most frequently used drugs in both the PENUT cohort and the general population were pulmonary medications (37% of EP infants, 8% of children in the general population). PENUT cohort children had significantly higher utilization of medications in every pulmonary subcategory. The most prevalent drug type in this category was inhaled beta agonists (15% of EP infants, 4% of children in the general population), followed by inhaled corticosteroids (13% in EP infants, 2% in the general population).

## 4. Discussion

In this study, we found that children born EP had higher rates of hospitalizations, procedures, medication use, and ED visits for comorbidities associated with prematurity compared to children of a similar age in the general population from 24 months to 59 months of age (Figure 1 and Figure 2; Table 2 and Table 3). Interestingly, children in the general population had a higher frequency of ED visits from 24 months to 59 months of age (Figure 1) as well as for common ailments such as gastrointestinal infections with dehydration and viral syndromes (Table 3), which may be due to decreased exposure to the healthcare system, with less established primary care as well as recognition of type of illness and severity.

Importantly, although EP infants may have had a higher baseline severity of illness as evidenced by increased hospitalizations compared to the general population (Table 2), they had a lower incidence of ED visits compared to the general population (Figure 1). These findings may be due to increased caregiver exposure to illness, improved hospital-to-home support with established primary care, and increased experience navigating the healthcare system. Preterm infants have a high number of outpatient follow-up visits after NICU discharge [6], which may allow for earlier detection and management of illness in the outpatient setting and thus diminish acute care needs. Additionally, it has been shown that parents who have established primary care providers and improved understanding of illness severity have an increased ability to navigate the healthcare system that leads to a reduction in non-urgent emergency department use [15]. Furthermore, in this same cohort of preterm infants, those with severe medical comorbidities were more likely to utilize outpatient interventional therapies such as occupational, speech, and physical therapy, increasing caregiver exposure to the healthcare system [16]. Moreover, even among parents with limited health literacy, those with targeted child-specific chronic illness knowledge have improved outcomes and ability to access the healthcare system [17].

Our data builds on previous studies demonstrating that respiratory illness is the most common reason for rehospitalization and ED visits among preterm infants [4,7,8]. Remarkably, EP infants had a similar proportion of hospitalizations related to respiratory concerns compared to their counterparts in the general population (Table 2). This contrasts with prior research demonstrating that preterm infants are more likely to be rehospitalized for respiratory disease [18]. It is possible that PENUT cohort infants in our study initially experienced a higher frequency of hospitalizations that dissipated prior to 24 months or experienced a similar frequency [13,14] but greater severity of respiratory readmissions. For example, McLaurin et al. found that respiratory syncytial virus (RSV) admissions were more costly and more severe in preterm infants, with a higher proportion of infants admitted to the intensive care unit, increased need for mechanical ventilation, and longer length of stay compared to term infants [19]. EP infants’ higher utilization of the ED for reactive airway disease and increased use of respiratory medications, such as bronchodilators, inhaled corticosteroids, montelukast, and oral steroids (Table 3, Figure 2, Supplementary Table S2), is consistent with the prior literature [2,6,20]. The high burden of reactive airway disease in EP infants may reflect long-term consequences of severe BPD [21], the most common co-morbidity in the PENUT cohort.

The PENUT cohort, however, exhibited a higher frequency of hospitalizations and ED visits for procedures compared to the general population (Table 2 and Table 3). A large subset of EP infants required procedures related to medical technologies, such as gastrostomy tubes and tracheostomies (Table 2), reflecting ongoing complications and comorbidities from their initial NICU hospitalization. Our findings are consistent with previous studies demonstrating an association between medical complexity and the need for medical technology at discharge and increased healthcare utilization among NICU graduates [7,9,22]. Similarly, the EP population had higher rates of ED visits for neurology-associated concerns such as seizures and apnea (Table 3), which is consistent with the literature of increased seizure risk and disordered breathing in early childhood following preterm birth [23,24].

EP infants were 4 times as likely to report any medication use during the follow-up period and had increased utilization in almost all drug categories (Figure 2). PENUT cohort infants were more likely to require respiratory medications, immunosuppressants, pancrelipase, muscle relaxants, anti-epileptic drugs, anti-reflux drugs, stool softeners and stimulants, levothyroxine, diuretics, and certain anti-hypertensive drugs, underscoring the wide range of follow-up needs in this population. The high utilization of respiratory medications and anti-epileptic drugs among EP infants parallels the greater incidence of encounters for pulmonary conditions and seizures in this population, respectively (Table 2 and Table 3, Figure 2, Supplementary Table S2). While Levin and colleagues reported that differences in medication utilization between preterm and term infants resolved by 19 months after discharge [2], our data indicate that these discrepancies persist throughout early childhood. Indeed, other studies have shown similar trends to ours in school-aged children aged 5–8 years, where medication utilization is greatest in those born EP [20].

While this study provides valuable insights into the healthcare utilization of NICU EP graduates, there are several limitations to be acknowledged. First, parental self-reporting of healthcare utilization may introduce inaccuracies and recall bias, although parent recall was found to be a reliable source of information regarding children’s healthcare utilization [25]. There may be discrepancies in comparing parent-reported diagnoses in the PENUT cohort to ICD-10 codes in the KID and NEDS databases. Furthermore, although Black infant enrollment was similar to national databases (Supplementary Table S1), this population was underrepresented in our follow-up cohort compared to PENUT enrollees (Table 1). In Ponnapakkam et al.’s analysis of the PENUT cohort, no significant association was found between maternal race and outpatient therapy utilization [16]. However, structural racism and social determinants of health may play a role in other aspects of healthcare utilization [11]. A large population-based study in California demonstrated that both Black and Hispanic early preterm infants had higher rates of hospital readmissions, and that Black moderate to late preterm infants also had increased post-discharge mortality [11]. Therefore, underrepresentation of Black infants in our cohort may have led to underestimation of healthcare utilization and illness severity. Future studies prioritizing the inclusion of EP infants from diverse backgrounds to better understand and address the profound racial disparities in preterm birth and healthcare outcomes after discharge are needed [11,26,27]. Furthermore, aside from ethnic and demographic data, other perinatal factors such as comorbidities or maternal factors such as education were not able to be compared between the PENUT cohort and the general population due to the availability of information in national datasets, presenting a limitation to this study. Patients in the PENUT cohort had inconsistent follow-up throughout the 24–60 month post-discharge period, making it difficult to draw definitive conclusions regarding longitudinal healthcare utilization in this group. In addition, the generalizability of a cohort derived from a randomized control study may have limitations when it comes to healthcare utilization due to systematic differences shown in consenting and inclusion of all demographics [28]. We also acknowledge that one limitation of the study is the large number of comparisons being made between the PENUT cohort and population databases. Therefore, we run the risk of type I error. Therefore, it is likely that some of the possible relationships highlighted have been identified purely by chance. However, as the purpose of the study was hypothesis generation with respect to ways in which being born EP may affect long-term health and healthcare utilization, we particularly wanted to avoid type II error caused by overcorrecting for multiple comparisons. Due to the convenience nature of the available datasets and the number of comparisons, we did not perform post hoc power calculations. However, the datasets deployed are some of the largest available datasets of their kind, so any true differences that exist between the populations are likely to have been detected. Finally, we also have to acknowledge that we were only able to perform raw comparisons across cohorts due to discrepancies in how potential confounders were coded. Therefore, we acknowledge that any findings may be subject to residual confounding.

Strengths of this study include the evaluation of a large, multi-site cohort of EP infants and accessing three large national databases to make comparisons to the general population in three domains of healthcare utilization: hospitalizations, ED visits, and medication use. Furthermore, this is one of the largest modern studies to follow up a group of EP infants with granular detail, allowing a meaningful contribution for generating hypotheses around healthcare utilization as well as highlighting the need to address disparities in follow-up. Additionally, data on hospital readmissions and ED visits among EP infants can contribute to the development and individualization of follow-up programs and longitudinal care planning for these patients.

## 5. Conclusions

In conclusion, EP infants had higher rates of overall hospitalizations, procedures, medication usage, and ED visits for concerns that may be linked to their pre-existing conditions. However, ED visits were less likely in the EP population overall and for common etiologies in this age range including dermatologic, gastrointestinal, and infectious disease concerns. This may be due to improved access and navigation of the healthcare system by accessing primary care as well as higher medical literacy regarding severity of illness.

## Figures and Tables

**Table 1:** Baseline patient characteristics for the PENUT cohort who survived to discharge compared to those with follow-up recorded between 24 and 60 months.

	PENUT Cohort Survived to Discharge n (%)	PENUT Cohort With Follow-Up 24–60 Months n (%)	*p*-Value
**Total (n)**	828	569	
**Maternal Information**			
Age (mean, SD)	28.8 (6.1)	29.3 (6.0)	***p* = 0.008**
Race			
Black	213 (25.7)	112 (19.7)	***p* = 0.009**
White	535 (64.6)	401 (70.5)	***p* = 0.022**
Other/Unknown	80 (9.7)	56 (9.8)	*p* = 0.911
Hispanic Ethnicity	174 (21.0)	127 (22.3)	*p* = 0.560
Maternal Education Level			
High School or Less	277 (33.5)	166 (29.2)	*p* = 0.091
Some College	254 (30.7)	176 (30.9)	*p* = 0.919
College Degree or Greater	207 (25.0)	162 (28.5)	*p* = 0.148
Not Reported	90 (10.9)	65 (11.4)	*p* = 0.746
Maternal Marital Status—Married	425 (51.3)	321 (56.4)	*p* = 0.061
**Neonatal Admission Data**			
Birth Weight in Grams (IQR)	810 (683–950)	810 (683–950)	*p* = 0.524
Gestational Age in Weeks (IQR)	26 (25–27)	26 (25–27)	*p* = 0.611
Antenatal Steroids Given	754 (91.1)	523 (91.9)	*p* = 0.576
Prenatal Care	789 (95.3)	548 (96.3)	*p* = 0.356
Respiratory Support at Birth	828 (100)	569 (100)	*p* = 1.000
Small for Gestational Age	118 (14.3)	80 (14.1)	*p* = 0.920
Treatment with Erythropoietin	418 (50.5)	295 (51.8)	*p* = 0.656
**Neonatal Comorbidities**			
Severe BPD *	534 (64.5)	374 (65.7)	*p* = 0.634
Severe NEC †	37 (4.5)	25 (4.4)	*p* = 0.947
Severe IVH ⁑	91 (11.0)	62 (10.9)	*p* = 0.956
Hydrocephalus	52 (6.3)	30 (5.3)	*p* = 0.431
Severe ROP ‡	67 (8.1)	50 (8.8)	*p* = 0.645
≥2 of the above	157 (19.0)	108 (19.0)	*p* = 0.993
≥3 of the above	44 (5.3)	28 (4.9)	*p* = 0.744
**Discharge Support**			
Any Home Oxygen/Respiratory Support	318 (38.4)	224 (39.4)	*p* = 0.717
Tracheostomy/Ventilated	15 (1.8)	7 (1.2)	*p* = 0.391
Gastrostomy Tube	80 (9.7)	49 (8.6)	*p* = 0.505

* BPD—bronchopulmonary dysplasia; † NEC—necrotizing enterocolitis; ⁑ IVH—intraventricular hemorrhage. ‡ ROP—retinopathy of prematurity. ***p* values in bold** are significant.

**Table 2:** Total number of hospitalizations by cause for PENUT infants with follow-up between 24 and 60 months and for the general pediatric population between ages 24 and 59 months. n = number of encounters; significance denoted by *p* < 0.05% denotes proportion of admissions by cause. General population data obtained from the 2019 Kids’ Inpatient Database (KID).

Cause of Admission	PENUT Cohort Overnight Hospitalizations n =152 Encounters 24–60 Months	General Population Overnight Hospitalizations n = 177,232 Encounters 24–59 Months	*p*-Value
Respiratory: Total	57 (37.5%)	61,005 (35.9%)	*p* = 0.681
Reactive Airway Disease	19 (12.5%)	14,819 (8.4%)	*p* = 0.065
Viral URI */Bronchiolitis/Pneumonia	36 (23.7%)	29,715 (16.8%)	***p* = 0.023**
Interstitial Lung Disease	0 (0.0%)	146 (0.0%)	*p* = 1.000
Upper Airway Concern (Larynx, Nasal Passages)	1 (0.7%)	352 (0.2%)	*p* = 0.204
Respiratory NOS †	1 (0.7%)	16,187 (9.1%)	***p* < 0.001**
Procedures: Total	52 (34.2%)	19,890 (11.7%)	***p* < 0.001**
Congenital Heart Disease Procedure/Cardiac Cath	5 (3.3%)	3608 (2.0%)	*p* = 0.274
Tracheostomy Malfunction/Revision/Removal	7 (4.6%)	538 (0.3%)	***p* < 0.001**
ENT ⁑ Procedure	18 (11.8%)	4344 (2.5%)	***p* < 0.001**
Dental	1 (0.7%)	222 (0.1%)	*p* = 0.064
Neuro Procedure	7 (4.6%)	3209 (1.8%)	***p* = 0.010**
GT/NGT ‡ Placement, Malfunction, or Infection	5 (3.3%)	1914 (1.1%)	***p* = 0.009**
Hernia Repair, Hip Dislocation, Talipes Equinovarus	6 (4.0%)	2519 (1.4%)	***p* = 0.009**
Renal Procedure	3 (2.0%)	1035 (0.6%)	***p* = 0.025**
Gastrointestinal: Total	18 (11.8%)	24,067 (14.2%)	*p* = 0.412
Constipation/Abdominal Distension	4 (2.6%)	3953 (2.2%)	*p* = 0.738
Dehydration	10 (6.6%)	5996 (3.4%)	***p* = 0.029**
Nutrition/Failure to Thrive	0 (0.0%)	1197 (0.7%)	*p* = 0.309
Liver/Biliary Tract/Pancreas	0 (0.0%)	594 (0.3%)	*p* = 0.475
GERD ⁂/Aspiration	0 (0.0%)	799 (0.5%)	*p* = 0.407
Surgical Abdomen and Post-op Complication	0 (0.0%)	3916 (2.2%)	*p* = 0.064
Infections/Inflammation	4 (2.6%)	4359 (2.5%)	*p* = 0.891
Imaging	0 (0.0%)	2505 (1.4%)	*p* = 0.140
Neurology: Total	9 (5.9%)	11,570 (6.8%)	*p* = 0.664
Seizures/Possible Seizures	7 (4.6%)	9637 (5.4%)	*p* = 0.531
Apnea	2 (1.3%)	151 (0.1%)	***p* < 0.001**
Dizziness/Headache	0 (0.0%)	186 (0.1%)	*p* = 0.689
Altered Mental Status/Encephalopathy	0 (0.0%)	318 (0.2%)	*p* = 0.601
Motor Disorders	0 (0.0%)	159 (0.1%)	*p* = 0.712
Neuroimaging	0 (0.0%)	996 (0.6%)	*p* = 0.354
Infectious Disease: Total	4 (2.6%)	16,913 (10.0%)	***p* = 0.003**
Fever Alone	1 (0.7%)	812 (0.5%)	*p* = 0.716
ENT Infections	0 (0.0%)	1852 (1.0%)	*p* = 0.205
Urinary Tract Infection	0 (0.0%)	2445 (1.4%)	*p* = 0.145
Viral Syndrome (Non- respiratory)	0 (0.0%)	2674 (1.5%)	*p* = 0.127
Sepsis/Meningitis	0 (0.0%)	4903 (2.8%)	***p* = 0.038**
Vaccination	0 (0.0%)	643 (0.4%)	*p* = 1.000
Other (Abscess, Periorbital Cellulitis, IV Antibiotics)	3 (2.0%)	3326 (1.9%)	*p* = 0.921
Accidental: Total	5 (3.3%)	7350 (4.3%)	*p* = 0.530
Trauma/Drowning	4 (2.6%)	4823 (2.7%)	*p* = 0.946
Ingestion: Toxins/Poisons	1 (0.7%)	1846 (1.0%)	*p* = 0.641
Foreign Body	0 (0.0%)	466 (0.3%)	*p* = 0.527
Child Neglect/Abuse	0 (0.0%)	452 (0.3%)	*p* = 0.533
Allergy: Total	1 (0.7%)	341 (0.2%)	*p* = 0.208
Environmental Allergy	0 (0.0%)	3 (0.0%)	*p* = 0.960
Acute Allergic Reaction	1 (0.7%)	312 (0.2%)	*p* = 0.157
Dermatology	0 (0.0%)	6245 (3.5%)	***p* = 0.019**
Endocrine	0 (0.0%)	2282 (1.3%)	*p* = 0.159
Hematology/Oncology	1 (0.7%)	17,349 (9.8%)	***p* < 0.001**
Renal	0 (0.0%)	1332 (0.8%)	*p* = 0.283
Psychiatric/Behavioral	0 (0.0%)	25 (0.0%)	*p* = 0.884
Rheumatologic/Autoimmune	0 (0.0%)	1159 (0.65%)	*p* = 0.317
Cardiac/Vascular/Lymphatic	0 (0.0%)	353 (0.2%)	*p* = 0.581
Metabolic/Genetic	0 (0.0%)	147 (0.1%)	*p* = 0.722

* URI—upper respiratory infection; † Respiratory NOS: Not Otherwise Specified (NOS) was ICD 10 codes of respiratory symptoms such as respiratory distress or failure without underlying etiology coded; ⁑ ENT—ear, nose, and throat; ‡ GT/NGT—gastrostomy tube/nasogastric tube. ⁂ GERD—gastroesophageal reflux disease. ***p* values in bold** are significant.

**Table 3:** Total number of ED visits by cause for PENUT infants with follow-up between 24 and 60 months and for the general pediatric population between ages 24 and 59 months. n = number of encounters; significance denoted by *p* < 0.05% denotes proportion of admissions by cause. General population data obtained from the 2019 National Emergency Department Sample (NEDS) database.

Cause of Visit to Emergency Department	PENUT Cohort Emergency Department Visits n = 402 Encounters 24–60 Months	General Population Emergency Department Visits n = 1,114,012 Encounters 24–59 Months	*p*-Value
**Respiratory: Total**	142 (35.0%)	325,706 (29.2%)	***p* = 0.010**
Reactive Airway Disease	48 (11.9%)	35,619 (3.2%)	***p* < 0.001**
Viral URI */Bronchiolitis/Pneumonia	94 (23.4%)	256,939 (23.1%)	*p* = 0.926
Interstitial Lung Disease	0 (0.0%)	130 (0.0%)	*p* = 1.000
Upper Airway Concern (Larynx, Nasal Passages)	0 (0.0%)	5642 (0.5%)	*p* = 0.281
Respiratory NOS †	0 (0.0%)	27,376 (2.5%)	***p* = 0.003**
**Procedures: Total**	28 (7.0%)	5209 (0.5%)	***p* < 0.001**
Congenital Heart Disease Procedure/Cardiac Cath	0 (0.0%)	376 (0.0%)	*p* = 1.000
Tracheostomy Malfunction/Revision/Removal	8 (2.0%)	96 (0.0%)	***p* < 0.001**
ENT ⁑ Procedure	1 (0.2%)	916 (0.1%)	*p* = 0.768
Dental	2 (0.5%)	1072 (0.1%)	*p* = 0.074
Neuro Procedure (i.e., Shunt Revision)	5 (1.2%)	262 (0.0%)	***p* < 0.001**
GT/NGT ‡ Placement, Malfunction, or Infection	12 (3.0%)	1967 (0.1%)	***p* < 0.001**
Hernia Repair, Hip Dislocation, Talipes Equinovarus	0 (0.0%)	500 (0.0%)	*p* = 1.000
Renal Procedure	0 (0.0%)	20 (0.0%)	*p* = 1.000
**Gastrointestinal: Total**	20 (4.9%)	134,914 (12.1%)	***p* < 0.001**
Constipation/Abdominal Distension	13 (3.2%)	55,411 (5.0%)	*p* = 0.136
Dehydration	7 (1.7%)	50,574 (4.5%)	***p* = 0.020**
Poor Nutrition/Failure to Thrive	0 (0.0%)	165 (0.0%)	*p* = 1.000
Liver/Biliary Tract/Pancreas	0 (0.0%)	811 (0.0%)	*p* = 1.000
GERD ⁂/Aspiration	0 (0.0%)	1993 (0.2%)	*p* = 0.796
Surgical Abdomen and Post-op Complication	0 (0.0%)	415 (0.0%)	*p* = 1.000
Infections/Inflammation	0 (0.0%)	25,545 (2.3%)	***p* = 0.004**
**Neurology: Total**	29 (7.2%)	22,449 (2.0%)	***p* < 0.001**
Seizures/Possible Seizures	24 (6.0%)	17,136 (1.5%)	***p* < 0.001**
Apnea	2 (0.5%)	143 (0.0%)	***p* < 0.001**
Dizziness/Headache	3 (7.5%)	3923 (0.4%)	*p* = 0.362
Altered Mental Status/Encephalopathy	0 (0.0%)	750 (0.1%)	*p* = 1.000
Motor Disorders	0 (0.0%)	482 (0.0%)	*p* = 1.000
Neuroimaging	0 (0.0%)	15 (0.0%)	*p* = 1.000
**Infectious Disease: Total**	87 (21.6%)	298,377 (26.8%)	***p* = 0.023**
Fever Alone	18 (4.5%)	55,822 (5.0%)	*p* = 0.707
ENT Infections	45 (11.2%)	147,883 (13.3%)	*p* = 0.248
Urinary Tract Infection	9 (2.2%)	16,580 (1.5%)	*p* = 0.300
Viral Syndrome (Non-respiratory)	11 (2.7%)	69,687 (6.3%)	***p* = 0.005**
Sepsis/Meningitis	0 (0.0%)	1610 (0.1%)	*p* = 0.916
Vaccination	0 (0.0%)	0 (0.0%)	*p* = 1.000
Other (Abscess, Periorbital Cellulitis, IV Antibiotics)	4 (1.0%)	6795 (0.6%)	*p* = 0.502
**Accidental: Total**	73 (18.2%)	242,415 (21.8%)	*p* = 0.091
Trauma/Drowning	71 (17.7%)	207,682 (18.6%)	*p* = 0.659
Ingestion: Toxins/Poisons	2 (0.5%)	11,778 (1.1%)	*p* = 0.393
Foreign Body	0 (0.0%)	22,068 (19.8%)	***p* = 0.008**
Child Neglect/Abuse	0 (0.0%)	887 (0.1%)	*p* = 1.000
**Allergy: Total**	11 (2.7%)	14,447 (1.3%)	***p* = 0.020**
**Environmental Allergy**	0 (0.0%)	2963 (0.3%)	*p* = 0.582
**Acute Allergic Reaction**	11 (2.7%)	11,484 (1.0%)	***p* = 0.002**
**Dermatology**	4 (1.0%)	60,838 (5.5%)	***p* < 0.001**
**Rheumatologic/Autoimmune**	0 (0.0%)	794 (0.1%)	*p* = 1.000
**Endocrine**	0 (0.0%)	1343 (0.1%)	*p* = 1.000
**Hematology/Oncology**	2 (0.5%)	3723 (0.3%)	*p* = 0.890
**Renal**	0 (0.0%)	692 (0.06%)	*p* = 1.000
**Psychiatric/Behavioral**	0 (0.0%)	102 (0.0%)	*p* = 1.000
**Cardiac/Vascular/Lymphatic**	0 (0.0%)	1328 (0.1%)	*p* = 1.000
**Metabolic/Genetic**	0 (0.0%)	31 (0.0%)	*p* = 1.000

* URI—upper respiratory infection; † Respiratory NOS: Not Otherwise Specified (NOS) was ICD 10 codes of respiratory symptoms such as respiratory distress or failure without underlying etiology coded; ⁑ ENT—ear, nose, and throat; ‡ GT/NGT—gastrostomy tube/nasogastric tube. ⁂ GERD—gastroesophageal reflux disease ***p* values in bold** are significant.

## Data Availability

The raw data supporting the conclusions of this article will be made available by the authors on request.

## Supplementary Materials

The following supporting information can be downloaded at: https://www.mdpi.com/article/10.3390/children12080979/s1, Table S1: Racial and ethnic demographic data for 2-, 3-, and 4-year-old patients in the PENUT cohort and general population; Table S2: Medication use in PENUT participants with follow-up between 24–60 months and in the general pediatric population between ages 24–59 months. n = number of encounters, significance denoted by *p* < 0.05, % denotes percentage of encounters with medication used. General population data from 2019 National Health and Nutrition Examination Survey (NHANES).

## Abbreviations

EP: extremely preterm; ED: emergency department; neonatal intensive care unit (NICU); Preterm Erythropoietin Neuroprotection (PENUT); gestational age (GA); Kids’ Inpatient Database (KID); Nationwide Emergency Department Sample (NEDS); National Health and Nutrition Examination Survey (NHANES); bronchopulmonary dysplasia (BPD); necrotizing enterocolitis (NEC); intraventricular hemorrhage (IVH); retinopathy of prematurity (ROP); International Classification of Diseases, Tenth Revision (ICD-10); upper respiratory infection (URI); Not Otherwise Specified (NOS); ear, nose, and throat (ENT); gastrostomy tube/nasogastric tube (GT/NGT); gastroesophageal reflux disease (GERD); respiratory syncytial virus (RSV).
