# Supplementary material for: Childhood Differences in Healthcare Utilization Between Extremely Preterm Infants and the General Population
Source: Children (Basel). 2025 Jul 25;12(8):979. doi: 10.3390/children12080979 (PMC12385164; doi:10.3390/children12080979)
Supplement: Supplementary file 1 [file children-12-00979-s001.zip › children-3731654-supplementary.pdf]

Supplemental Table S1. Racial and ethnic demographic data for 2-, 3-, and 4-year-old patients in the PENUT cohort and general population.

	PENUT (Original) n (%)	PENUT (Recoded) n (%)	KID n (%)	NEDS n (%)	NHANES n (%)
Total (n)	569	569	166,323	1,114,012	3,600
Race					
White*	401 (70.5%)	296 (52.0%)	71,506 (43.0%)	433,106 (38.9%)	1,056 (29.3%)
Black*	112 (19.7%)	108 (19.0%)	27,901 (16.8%)	224,036 (20.1%)	864 (24.0%)
Other or Unknown	56 (9.8%)	54 (6.7%)	26,445 (15.9%)	172,243 (15.4%)	574 (15.9%)
Ethnicity					
Hispanic*	127 (22.3%)	127 (22.3%)	40,471 (24.3%)	284,627 (25.5%)	1,106 (30.7%)

* In KID, NEDS, and NHANES, race/ethnicity was coded as Hispanic, non-Hispanic Black, or non-Hispanic white. In PENUT, white and Black racial identity were classified separately from Hispanic ethnicity. The first column shows the data as originally coded in PENUT. The second column shows the PENUT data recoded to make Hispanic a separate category to align with the other datasets. Across all four datasets, the proportion of non-Hispanic white individuals compared to other categories combined was significantly different (p<0.0001, Chi-square test).

Supplemental Table S2. Medication use in PENUT participants with follow-up between 24-60 months and in the general pediatric population between ages 24-59 months. n= number of encounters, significance denoted by p <0.05, % denotes percentage of encounters with medication used. General population data from 2019 National Health and Nutrition Examination Survey (NHANES).

Medication	PENUT Cohort Medication Use n = 1319 encounters 24-60 months	General Population Medication Use n = 3600 encounters 24-59 months	p-value
**Allergy/Immunology: Total**	82 (6.2%)	117 (3.2%)	**p < 0.001**
**Oral Antihistamine**	50 (3.8%)	49 (1.4%)	**p < 0.001**

**Epinephrine**	2 (0.2%)	2 (0.1%)	p = 0.294
**Topical Steroid**	8 (0.6%)	41 (1.1%)	p = 0.097
**Nasal Corticosteroid**	18 (1.4%)	22 (0.6%)	**p = 0.009**
**Antihistamine Ophthalmic/Nasal**	0 (0.0%)	3 (0.1%)	p = 0.295
**Immunosuppressant**	4 (0.3%)	0 (0.0%)	**p < 0.001**
**Neurology: Total**	81 (6.1%)	24 (0.7%)	**p < 0.001**
**Baclofen/Botox**	12 (0.9%)	4 (0.1%)	**p < 0.001**
**Benzodiazepine**	3 (0.2%)	3 (0.1%)	p = 0.199
**Clonidine**	3 (0.2%)	6 (0.2%)	p = 0.657
**Gabapentin**	1 (0.1%)	0 (0.0%)	p = 0.098
**Levetiracetam**	34 (2.6%)	7 (0.2%)	**p < 0.001**
**Other Anti-Epileptic Medications**	15 (1.1%)	4 (0.1%)	**p < 0.001**
**Melatonin**	13 (1.0%)	0 (0.0%)	**p < 0.001**
**Gastrointestinal: Total**	144 (10.9%)	39 (1.1%)	**p < 0.001**
**Proton Pump Inhibitor**	46 (3.5%)	4 (0.1%)	**p < 0.001**
**H2 Blocker**	28 (2.1%)	6 (0.2%)	**p < 0.001**
**Ondansetron**	4 (0.3%)	11 (0.3%)	p = 0.992
**Pancrelipase**	3 (0.2%)	0 (0.0%)	**p = 0.004**
**Stool Softeners/Stimulants**	63 (4.8%)	18 (0.5%)	**p < 0.001**
**Pulmonary: Total**	483 (36.6%)	293 (8.1%)	**p < 0.001**
**Inhaled Beta Agonist**	202 (15.3%)	153 (4.2%)	**p < 0.001**
**Inhaled Beta Agonist + Corticosteroid**	16 (1.2%)	5 (0.1%)	**p < 0.001**
**Inhaled Corticosteroid**	165 (12.5%)	77 (2.1%)	**p < 0.001**

**Inhaled Beta Agonist + Ipratropium**	5 (0.4%)	0 (0.0%)	**p < 0.001**
**Ipratropium**	3 (0.2%)	0 (0.0%)	**p = 0.004**
**Montelukast**	29 (2.2%)	34 (0.9%)	**p < 0.001**
**Oral Steroid**	34 (2.6%)	22 (0.6%)	**p < 0.001**
**Respiratory Inhalant Products - Unspecified**	7 (0.5%)	2 (0.1%)	**p < 0.001**
**Pulmonary Vasodilators**	22 (1.7%)	0 (0.0%)	**p < 0.001**
**Renal/Cardiovascular: Total**	28 (2.1%)	6 (0.2%)	**p < 0.001**
**Thiazide**	5 (0.4%)	0 (0.0%)	**p < 0.001**
**Loop Diuretic**	6 (0.5%)	1 (0.0%)	**p < 0.001**
**Spironolactone**	3 (0.2%)	0 (0.0%)	**p = 0.004**
**Enalapril**	0 (0.0%)	2 (0.1%)	p = 0.392
**Calcium Chanel Blocker**	11 (0.8%)	1 (0.0%)	**p < 0.001**
**Losartan**	3 (0.2%)	0 (0.0%)	**p = 0.004**
**Beta Blocker**	0 (0.0%)	2 (0.1%)	p = 0.392
**Endocrine: Total**	16 (1.2%)	7 (0.2%)	**p < 0.001**
**Levothyroxine**	12 (0.9%)	5 (0.1%)	**p < 0.001**
**Fludrocortisone**	2 (0.2%)	0 (0.0%)	p = 0.019
**Sex Hormones - Unspecified**	1 (0.1%)	0 (0.0%)	p = 0.098
**Somatropin**	0 (0.0%)	2 (0.1%)	p = 0.392
**Megestrol**	1 (0.1%)	0 (0.0%)	p = 0.098
**Psychiatric/Behavioral: Total**	2 (0.2%)	3 (0.1%)	p = 0.504
**Stimulant**	0 (0.0%)	2 (0.1%)	p = 0.392
**Imipramine**	0 (0.0%)	1 (0.0%)	p = 0.545

**Guanfacine**	2 (0.2%)	0 (0.0%)	p = 0.019
**Other: Total**	17 (1.3%)	2 (0.1%)	
**Acetazolamide**	1 (0.1%)	0 (0.0%)	p = 0.098
**Aspirin**	2 (0.2%)	0 (0.0%)	p = 0.019
**Oxybutynin**	2 (0.2%)	0 (0.0%)	p = 0.019
**Tamsulosin**	3 (0.2%)	1 (0.0%)	p = 0.029
**Atropine Ophthalmic**	1 (0.1%)	1 (0.0%)	p = 0.458
**Medication Use By # of Comorbidities**	Any Medication Use (%)		
**0 (n =167)**	72 (43.1%)		
**1 (n = 291)**	173 (59.5%)		
**2 (n =80)**	80 (67.5%)		
**≥ 3 (n =28)**	16 (57.1%)		
